# Effects of Nutrient Intake Timing on Post-Exercise Glycogen Accumulation and Its Related Signaling Pathways in Mouse Skeletal Muscle

**DOI:** 10.3390/nu11112555

**Published:** 2019-10-23

**Authors:** Yumiko Takahashi, Yutaka Matsunaga, Mai Banjo, Kenya Takahashi, Yosuke Sato, Kohei Seike, Suguru Nakano, Hideo Hatta

**Affiliations:** Department of Sports Sciences, The University of Tokyo, 3-8-1 Komaba, Meguro-ku, Tokyo 153-8902, Japan; y_matsunaga@idaten.c.u-tokyo.ac.jp (Y.M.); mai-banjo@lime.ocn.ne.jp (M.B.); aynekihsahakat@gmail.com (K.T.); amusnsd@gmail.com (Y.S.); kseike302043j@gmail.com (K.S.); s.tion@icloud.com (S.N.); hatta@idaten.c.u-tokyo.ac.jp (H.H.)

**Keywords:** skeletal muscle, glycogen, post-exercise, Akt, AS160, nutrient intake

## Abstract

We investigated the effects of nutrient intake timing on glycogen accumulation and its related signals in skeletal muscle after an exercise that did not induce large glycogen depletion. Male ICR mice ran on a treadmill at 25 m/min for 60 min under a fed condition. Mice were orally administered a solution containing 1.2 mg/g carbohydrate and 0.4 mg/g protein or water either immediately (early nutrient, EN) or 180 min (late nutrient, LN) after the exercise. Tissues were harvested at 30 min after the oral administration. No significant difference in blood glucose or plasma insulin concentrations was found between the EN and LN groups. The plantaris muscle glycogen concentration was significantly (*p* < 0.05) higher in the EN group—but not in the LN group—compared to the respective time-matched control group. Akt Ser^473^ phosphorylation was significantly higher in the EN group than in the time-matched control group (*p* < 0.01), while LN had no effect. Positive main effects of time were found for the phosphorylations in Akt substrate of 160 kDa (AS160) Thr^642^ (*p* < 0.05), 5′-AMP-activated protein kinase (AMPK) Thr^172^ (*p* < 0.01), and acetyl-CoA carboxylase Ser^79^ (*p* < 0.01); however, no effect of nutrient intake was found for these. We showed that delayed nutrient intake could not increase muscle glycogen after endurance exercise which did not induce large glycogen depletion. The results also suggest that post-exercise muscle glycogen accumulation after nutrient intake might be partly influenced by Akt activation. Meanwhile, increased AS160 and AMPK activation by post-exercise fasting might not lead to glycogen accumulation.

## 1. Introduction

Glycogen stored in skeletal muscle is the predominant energy source for muscle contraction when the exercise intensity is moderate to high in humans [1,2,3]. Skeletal muscle glycogen has been suggested to play a role in force production by modulating the excitation–contraction coupling process [4]. Therefore, the glycogen level in skeletal muscle is considered as a major determinant of exercise performance. After exercise, skeletal muscle glycogen repletion should be a primary goal in the preparation for subsequent training/competitive games. In many sport nutrition guidelines, carbohydrate and protein intake is recommended as the most effective means of post-exercise glycogen accumulation, because these nutrients increase the availability of glucose, a primary source of glycogen synthesis, and stimulate the secretion of insulin, a hormone-activating glucose uptake and glycogen synthesis [5,6,7].

Ivy et al. found that carbohydrate intake immediately after exercise resulted in a higher skeletal muscle glycogen accumulation rate in comparison with intake at 2 h after exercise in humans [8]. A later study showed that net uptake of glucose in the legs was higher when nutrients, including carbohydrate and protein, were ingested immediately after exercise than when they were ingested 3 h after exercise [9]. Although a delayed (2 h) carbohydrate intake did not affect the skeletal muscle glycogen level at >8 h after exercise [10], nutrient intake timing would be expected to have some influence when the recovery time from exercise is restricted. In most of the studies examining the influence of nutrient timing on glycogen recovery, the exercise was performed to exhaustion [8,10] or after an overnight fast [8,9,10] in order to deplete glycogen stored in the skeletal muscle. However, such severe glycogen-depletion protocols are not always practical for daily exercise. Meanwhile, successive days of exercise which alone do not deplete skeletal muscle glycogen storage may gradually decrease glycogen level [11,12]. Thus, investigation of the effects of nutrient intake timing on glycogen restoration after exercise without large glycogen depletion are also needed.

Important steps for glycogen synthesis are glucose uptake and the incorporation of glycosyl residue from uridine diphosphate glucose into glycogen catalyzed by glycogen synthase (GS) [13,14]. In general, exercise (i.e., skeletal muscle contraction) stimulates glucose uptake and GS activation without the requirement of insulin for 30 min to 2 h [15,16,17,18]. After that, insulin-dependent glucose uptake and glycogen synthesis are enhanced [16,17,19] for at least several hours [14]. The GS activity is regulated by dephosphorylation at several regulatory sites and by the allosteric activator glucose-6-phosphate (G6P) [20,21,22]. Glucose uptake would influence glycogen synthesis by supplying not only the substrate but also the allosteric activator for GS. In vitro studies have reported that insulin-stimulated glucose uptake is enhanced during the post-exercise phase in association with an increase in the phosphorylation of the Akt substrate of 160 kDa (AS160) [23,24,25]. AS160 is regulated by both an insulin-mediated pathway via Akt activation and a contraction-mediated pathway via 5′ AMP-activated protein kinase (AMPK) activation [26]. Several previous studies indicated that phosphorylation of AS160 in response to insulin stimulation was additively increased by exercise compared to that in a sedentary group at 3–4 h after exercise in isolated skeletal muscle [23,24,25,27]. While many studies have investigated the phosphorylation levels of signaling molecules involved in post-exercise glycogen synthesis with in vitro experimental models or hyperinsulinemic euglycemic clamp, the relationship between post-exercise glycogen accumulation and activation of signaling molecules after nutrient intake has not been fully investigated, and a comparison between different nutrient intake timings has not been undertaken.

In the present study, we investigated the influence of nutrient intake timing on glycogen accumulation in skeletal muscle and its related signaling pathways after endurance exercise that did not induce severe glycogen depletion. We provided mice with a nutrient solution containing carbohydrate and protein either immediately after or at 180 min after the exercise. One of the benefits for using mice is that their diet can be controlled easily. Dietary habits sometimes influence on protein expressions and enzymatic activities related to metabolism in skeletal muscle. As the entire muscle of mice could be used for analysis because of small size, it is also beneficial for analyzing without variation due to cutting off the samples. Previous studies showed that significant glycogen accumulation could be observed in mice skeletal muscle from both exhaustive [28] and not exhaustive exercise [29] in response to nutrient intake.

## 2. Materials and Methods

### 2.1. Animals

Eight-week-old male ICR mice were purchased from CLEA Japan (Tokyo). The mice were housed at four mice per cage in a room maintained at 23 °C, and allowed to acclimatize for 1 week. The mice were given free access to water and a standard chow (MF: 3.6 kcal/g, 60% kcal from carbohydrate, 13% kcal from fat, 27% kcal from protein; Oriental Yeast, Tokyo). All procedures involving animals in this study were conducted in accordance with the ethical standards of the Committee on Animal Care and Use, The University of Tokyo, and all protocols of research on animals were approved by this committee (approval no. 24-4). The dark phase for the mice was set as 07:00–19:00, and all experimental treatments were performed during the dark phase when the mice were active.

### 2.2. Experimental Procedures

All the mice were familiarized with the treadmill with shock grids (MK-680, Muromachi Kikai Co., Inc., Tokyo, Japan) exercise at a speed of 25 m/min for 10 min for 3 days. They were housed individually in standard cages. On the experimental day, after the mice were fasted for 1 h to avoid a postprandial state, they ran on the treadmill at 25 m/min for 60 min.

#### 2.2.1. Experiment 1

After treadmill running, the mice (*n* = 8) were orally administered 1.2 mg/g body weight (bw) carbohydrate (glucose) and 0.4 mg/g bw protein (milk casein) dissolved in water using a stomach tube (volume of ingestion: 0.01 ml/g bw). The amount of carbohydrate was expected to maximize the increase in skeletal muscle glycogen and the amount of protein was likely to stimulate insulin secretion [5,6]. Mice were orally administered solutions either immediately after treadmill running (early nutrient, EN) or at 180 min after treadmill running (late nutrient, LN). Blood samples were collected in heparinized capillary tubes from the tail vein before administration 0 and 15, 30 min after administration. All mice performed two experimental tests (early nutrition treatment and late nutrition treatment) as repeated measures with a randomized crossover design. The interval between the two experimental tests was set at 1 week. The heparinized capillary tubes were centrifuged and then plasma samples were collected and stored at −80 °C. The incremental areas under the curve (iAUC) for blood glucose and plasma insulin concentrations were calculated with the trapezoidal rule.

#### 2.2.2. Experiment 2

**Figure 1:**
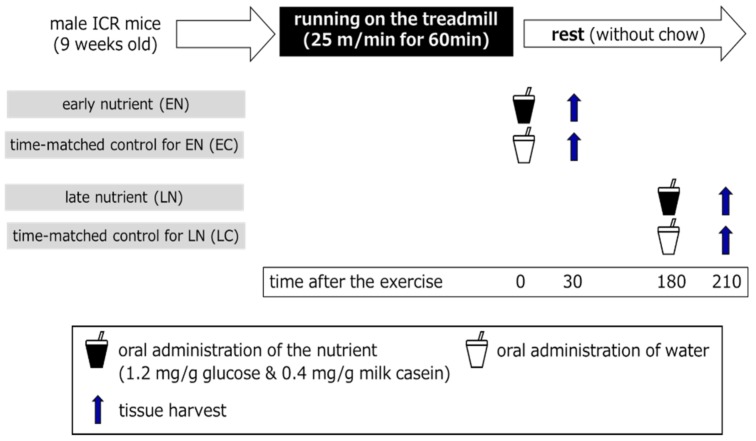
The design of experiment 2. Male ICR mice (9 weeks old) ran on a treadmill at 25 m/min for 60 min in a fed state. Mice were orally administered nutrients (early nutrient (EN), late nutrient (LN)) or water (EN group control (EC), LN group control (LC)) immediately after (EN, EC) or 180 min after (LN, LC) running. Tissues were harvested at 30 min after the oral administration. Chow was removed at 60 min before running to avoid a postprandial state and it was not provided during the post-exercise phase.

The mice with similar mean body weights were divided into five groups: a baseline (sedentary) group (*n* = 6), an early nutrient-treated (EN) group (*n* = 6), a group of controls time-matched to the EN group (EC, *n* = 7), a late nutrient (LN) group (*n* = 6), and a group of controls time-matched to the LN group (LC, *n* = 7). The design of experiment 2 was described in Figure 1. Mice in the nutrient-treated group were orally administered 1.2 mg/g bw glucose and 0.4 mg/g bw milk casein dissolved in water just as in experiment 1, while mice in the time-matched control group were orally administered water. Mice in the EC and EN groups were provided solutions immediately after the exercise, while mice in the LC and LN groups were provided solutions at 180 min after the exercise. At 30 min after the oral administration, mice were sacrificed under anesthesia and the tissues were harvested. Mice in the baseline group were provided water after 1 h of food removal and then sacrificed at 30 min after the water administration. The tissues were quickly frozen in liquid nitrogen and stored at −80 °C.

### 2.3. Analytical Methods

#### 2.3.1. Blood Analysis

Blood glucose concentrations were measured using an auto analyzer (Glutest Ace; Arkray Inc., Kyoto, Japan). Plasma insulin concentrations were measured using an enzyme-linked immunosorbent assay (ELISA) kit (Mouse Insulin ELISA Kit; Mercodia AB, Uppsala, Sweden). Plasma free fatty acid concentrations were measured using an assay kit (NEFA-C Wako; Wako, Tokyo, Japan).

#### 2.3.2. Glycogen Levels in Skeletal Muscle

The glycogen concentrations in the plantaris muscles and the liver were measured using the phenol-sulfuric acid method as described previously [30]. Plantaris muscle is one of the plantar flexors and recruited during treadmill running. In previous study, glycogen accumulation by glucose intake after treadmill running was observed in that muscle [29].

#### 2.3.3. Muscle Homogenization

Protein isolation from plantaris muscles was performed as described previously [31] in a radio-immunoprecipitation assay (RIPA) buffer (50 mM Tris-HCl pH 7.4, 150 mM NaCl, 0.25% deoxycholic acid, 1% NP-40, and 1 mM ethylenediaminetetraacetic acid (EDTA)) supplemented with a protease inhibitor mixture (cOmplete Mini, EDTA-free; Roche Applied Science, Mannheim, Germany), and a phosphatase inhibitor mixture (PhosSTOP; Roche Applied Science). After centrifugation at 600 *g* for 20 min at 4 °C, the supernatants were collected, and their protein concentrations were determined by a bicinchoninic acid assay (Thermo Fisher Scientific, Waltham, MA, USA). The supernatants were diluted with a RIPA buffer.

#### 2.3.4. Western Blotting

Western blotting was performed as described previously [31,32]. The protein samples (5–10 μg) and a pre-stained molecular weight marker (Bio-Rad, Hercules, CA, USA) were run on 7.5% sodium dodecyl sulfate-polyacrylamide gel electrophoresis (SDS-PAGE) gels for 60 min at 150 V. The proteins were then transferred from the gels to polyvinylidene difluoride (PVDF) membranes using Transblot Turbo (Bio-Rad). Next, the membranes were then blocked with PVDF blocking reagent (Toyobo, Osaka, Japan) for 60 min at room temperature. The membranes were incubated with the primary antibody in Can Get Signal Solution 1 (Toyobo) (1:1000 or 1:2000 dilution) overnight at 4 °C. They were then incubated for 60 min at room temperature with goat-anti-rabbit IgG (American Qualex, San Clemente, CA) in Can Get Signal Solution 2 (Toyobo) (1:5000 dilution). The proteins were detected using Pierce ECL Western Blotting Substrate (Thermo Fisher Scientific) and visualized using a ChemiDoc system (Bio-Rad). Densitometric analyses of the captured images were performed using Bio-Rad Quantity One software ver. 4.6.1. All the membranes were stained with Ponceau-S solution (P7170-1L; Sigma-Aldrich) to ensure equal loading of the proteins (data not shown).

The antibodies used in this study were anti-5′ AMP-activated protein kinase (AMPK, #5832; Cell Signaling Technology [CST] Japan, Tokyo), anti-phosphorylated AMPK (Thr172, #2535; CST), anti-acetyl-CoA carboxylase (ACC, #3676; CST), anti-phosphorylated ACC (Ser79, #11818; CST), Akt substrate 160 kDa (AS160, #2670; CST), phosphorylated AS160 (Thr642, #8881; CST, Ser588, #8730; CST), phosphorylated Akt (Thr308, #9275; CST, Ser473, #9271; CST), tre-2/USP6, BUB2, cdc16 domain family member 1 (TBC1D1, #2670; CST), phosphorylated TBC1D1 (Ser237, 07-2268; Millipore, Bedford, MA), glycogen synthase (GS, #3893; CST), and phosphorylated GS (Ser641, #3891; CST).

### 2.4. Statistical Analysis

All values are expressed as the mean ± standard error. Power analyses were not performed in the present study. Prism 7 software (GraphPad Software, San Diego, CA) was used for the statistical analysis. In experiment 1, two-way repeated analysis of variance (time × nutrition intake) was performed to analyze blood glucose and plasma insulin concentrations. The significance of differences in iAUCs was analyzed by repeated t-test. In experiment 2, two-way analysis of variance (time × nutrition intake) was performed to determine the differences in each parameter. If an interaction was observed, a Tukey–Kramer multiple comparison test was performed. Statistical significance was set at *p* < 0.05.

## 3. Results

### 3.1. Experiment 1

#### Blood Glucose and Plasma Insulin Levels after the Nutrient Intake

**Figure 2:**
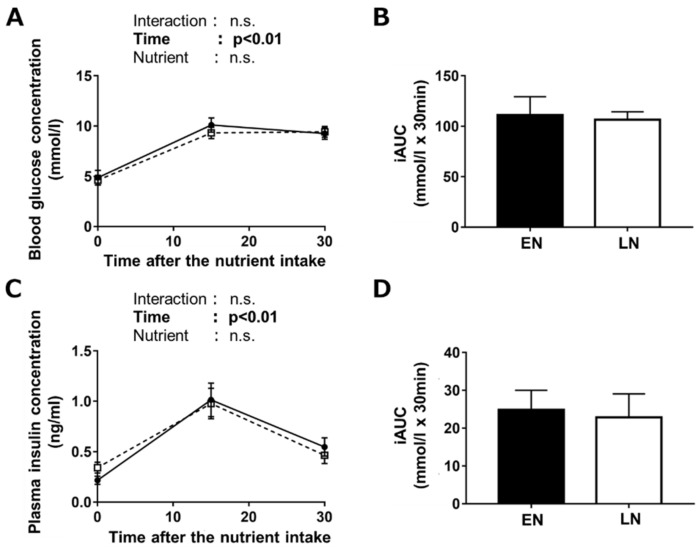
(**A**) Blood glucose and (**C**) plasma insulin concentrations after intake on an oral nutrient solution (1.2 mg/g bw glucose and 0.4 mg/g milk casein). The nutrient solution was orally administered immediately after the exercise (EN, black) or 180 min after the exercise (LN, white). The incremental areas under the curve (iAUCs) of blood glucose (**B**) and (**D**) plasma insulin concentrations are shown. Values are mean ± standard error (SE). *n* = 8 per group.

There was a significant main effect of time during the 30 min post-oral nutrient administration phase on blood glucose concentration (Figure 2A). No main effect of nutrient timing was found. There was no significant difference in the incremental area under the curve (iAUC) of blood glucose concentration after the nutrient treatment (Figure 2B). There was also no effect of nutrient timing on the plasma insulin concentration during the 30 min post-administration phase (Figure 2C), and no significant difference in iAUC was found (Figure 2D).

### 3.2. Experiment 2

#### 3.2.1. Glycogen Level in the Plantaris Muscle and Liver

**Figure 3:**
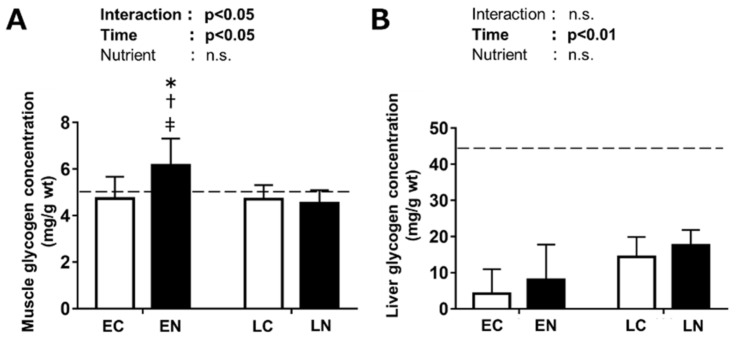
Mouse plantaris muscle (**A**) and liver (**B**) glycogen concentrations at 30 min after oral intake of nutrient solution (1.2 mg/g bw glucose and 0.4 mg/g milk casein, black) or water (control, white). The early nutrient-treated (EN) and the time-matched control (EC) groups were provided solution immediately after the exercise; the late nutrient-treated (LN) and the time-matched control (LC) groups were provided solution at 180 min after the exercise. Values are mean ± SE. *n* = 6 or 7 per group. Dashed lines indicate the baseline values (sedentary). * *p* < 0.05 vs. the EC, ^†^ *p* < 0.05 vs. the LC, ^‡^ *p* < 0.05 vs. the LN.

The plantaris muscle glycogen concentration in the baseline (sedentary) group was 5.1 ± 0.3 mg/g wet weight (wt). There was a significant interaction between nutrient treatment and time after the exercise (*p* < 0.05) (Figure 3A). We performed a Tukey–Kramer multiple-comparison test, and found that the plantaris muscle glycogen concentration in the EN group was significantly higher than that in the EC group (*p* < 0.05). On the other hand, no significant difference was found between glycogen levels in the LN and LC group. The glycogen concentration in the EN group was significantly higher than those in the LN and LC groups (*p* < 0.05).

The liver glycogen concentration in the baseline group was 42.7 ± 4.9 mg/g wt. There was a significant positive main effect of time after the exercise (*p* < 0.01, Figure 3B). However, no significant main effect of nutrient intake was found.

#### 3.2.2. Phosphorylation Levels of Signaling Pathway Proteins Related to Carbohydrate Metabolism

**Figure 4:**
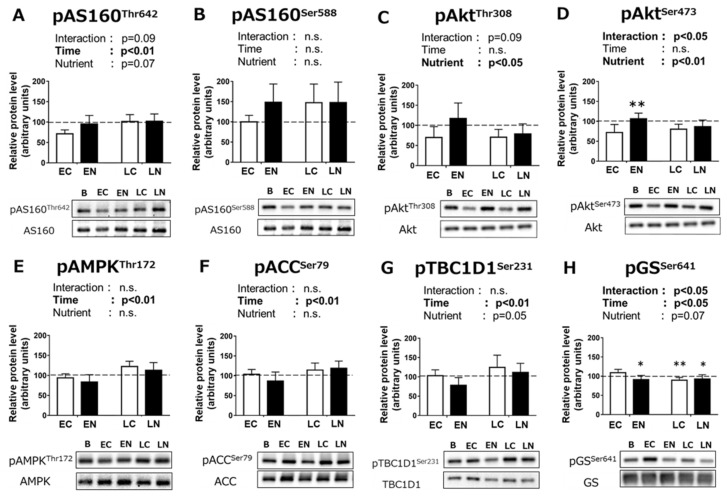
Effects of post-exercise nutrient intake on phosphorylation levels of proteins related to carbohydrate metabolism. (**A**) Akt substrate 160 kDa (AS160) Thr642 and (**B**) Ser588, (**C**) Akt Thr308 and (**D**) Ser473, (**E**) 5’-AMP-activated protein kinase (AMPK) Thr172, (**F**) acetyl-CoA carboxylase (ACC) Ser79, (**G**) TBC1 domain family member 1 (TBC1D1) Ser231, and (**H**) glycogen synthase (GS) Ser641 phosphorylation status in mouse plantaris muscle at 30 min after oral intake of nutrients or water. The experimental protocol was the same as that described in the Figure 3 legend. Dashed lines indicate the baseline values (B, sedentary control). All data shown are relative to the baseline values ± SE. *n* = 6 or 7 per group. * *p* < 0.05, ** *p* < 0.01 vs. the EC.

Increased phosphorylation of Akt substrate 160 kDa (AS160) has been suggested to be associated with the enhancement of insulin-stimulated glucose uptake during the post-exercise phase. In the present study, we observed a positive main effect of time on AS160 Thr^642^ phosphorylation (*p* < 0.01, Figure 4A). AS160 Thr^642^ phosphorylation tended to be higher in the nutrient-treated groups, but the main effect was not statistically significant (*p* = 0.07). No significant main effect of time or nutrient treatment was found in AS160 Ser^588^ phosphorylation (Figure 4B).

We measured the phosphorylation of Akt, which is known to be a central hub connecting insulin signaling with AS160 phosphorylation. Insulin stimulation induces Akt Thr^308^ and Ser^473^ phosphorylations. There was a positive main effect of nutrient intake on Akt Thr^308^ phosphorylation (*p* < 0.05, Figure 4C). A significant interaction between nutrient intake and time was observed in Akt Ser^473^ phosphorylation (*p* < 0.05, Figure 4D). The results of the multiple-comparison test showed that Akt Ser^473^ phosphorylation in the EN group was significantly higher than that in the EC group (*p* < 0.01), while no significant difference was found between the LC and LN groups.

We observed a significant positive main effect of time on the phosphorylated state of 5′ AMP-activated protein kinase (AMPK, *p* < 0.01; Figure 4E), an upstream kinase of AS160, but we did not observe a significant main effect of nutrient intake. Its phosphorylation is related to activation of AMPK. We also found a significant positive main effect of time on the phosphorylation level of acetyl-CoA carboxylase (ACC), the substrate for AMPK (*p* < 0.01), but we did not find a significant main effect of nutrient intake (Figure 4F).

TBC1D1 Ser^231^ is known to be phosphorylated by AMPK. There was a significant positive main effect of time on TBC1D1 Ser^231^ phosphorylation (*p* < 0.01, Figure 4G). TBC1D1 Ser^231^ phosphorylation after nutrient intake groups tended to be lower than that without nutrient intake groups (*p* = 0.05).

We also measured the phosphorylated state of glycogen synthase (GS) Ser^641^, one of the major regulatory sites of GS activity. Phosphorylation at this site is associated with the reduced fractional activity. There was a significant interaction between time and nutrient intake (*p* < 0.05, Figure 4G). The multiple-comparison test revealed that GS Ser^641^ phosphorylation was significantly lower in the EN, LC, and LN groups compared to the EC group (*p* < 0.05 or *p* < 0.01).

#### 3.2.3. Plasma Free Fatty Acid (FFA) Level

**Figure 5:**
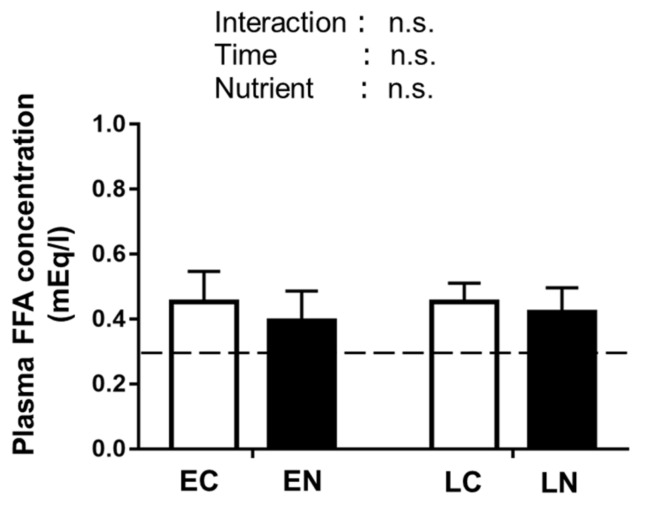
Plasma free fatty acid (FFA) concentrations in mice at 30 min after oral intake of nutrients or water. The experimental protocol was the same as that described in the Figure 3 legend. Dashed lines indicate the baseline values (B, sedentary control). Values are the mean ± SE. *n* = 6 or 7 per group.

Because glucose uptake and glycogen synthesis are inhibited when the plasma free fatty acid (FFA) level becomes higher [33,34,35], we measured the plasma FFA levels. No significant main effect of time or nutrient treatment was found in plasma FFA level at 30 min after the oral administration (Figure 5).

## 4. Discussion

In the present study, we investigated the effects of post-exercise nutrient (carbohydrate plus protein) intake timing on skeletal muscle glycogen accumulation and its related signaling pathways using mice. While previous studies revealing a relationship between nutrient intake timing and post-exercise glycogen recovery have used severe exercise protocols to deplete glycogen storage (e.g., exercise to exhaustion or overnight fasting) [8,9,10], in the present study we used moderate endurance exercise without prolonged fast. The protocol of treadmill running used in the present study was shown to have enough stimulation for increasing the maximal activity of citrate synthase, usually used as an indicator of mitochondrial oxidative capacity [36]. Plantaris muscle glycogen concentration after 30 min of exercise without post-exercise nutrient intake (EC group) seemed not to be lower than the baseline level. In rodents, skeletal muscle glycogen recovery after treadmill running seems to occur even in the absence of nutrient intake at 30–60 min after exercise [17,18]. Therefore, the possibility exists that glycogen was accumulated to some extent in the group without nutrient treatment over the first 30 min after exercise. Moreover, as the difference in liver glycogen between the EC group and the baseline group was greater than that in skeletal muscle glycogen, mice might use liver glycogen preferentially during the exercise. While the importance of skeletal muscle glycogen for exercise in mice has not reached a conclusion yet [37,38], mice seem to increase reliance on it as the exercise intensity becomes higher as well as human. For example, previous study reported that running exercise with higher speed (30 m/min) compared to that in the present study induced about 40% of glycogen reduction in skeletal muscle [39]. Other mouse study showed that an incremental running test induced large skeletal muscle glycogen depletion at exhaustion while liver glycogen was not depleted [40]. In the present study, even though the exercise did not induce a major depletion of skeletal muscle glycogen, the results showed that it provided enough stimulation for glycogen to accumulate in skeletal muscle in response to carbohydrate and protein intake in the period immediately after exercise. We found significant difference in skeletal muscle glycogen accumulation by different post-exercise nutrient intake timing. In the present study, both blood glucose and plasma insulin concentrations were similarly increased by the nutrient intake immediately after exercise and the nutrient intake at 180 min after exercise. However, although the glucose availability in blood and increase in insulin were similar to those in the group given nutrient treatment immediately after exercise, glycogen accumulation in the plantaris muscle did not occur after nutrient intake at 180 min after exercise. Similar results have been reported in previous human studies with large skeletal muscle glycogen depletion [8,9,10].

Next, we examined the influence of nutrient intake timing on the phosphorylation states of signaling molecules suggested to be involved in glucose uptake and glycogen synthesis. This study is the first in vivo study investigating effects of post-exercise nutrient intake timing on these phosphorylation states in skeletal muscle. AS160 is a distal signaling protein that plays a role in glucose uptake by stimulating glucose transporter (GLUT) 4 translocation. As in vitro studies have reported that enhanced insulin-stimulated glucose uptake after exercise is associated with an increase in the phosphorylation of the AS160 [23,24,25], we measured the phosphorylation levels of AS160. We found main positive effects of 180 min fast after the exercise on AS160 Thr^642^ phosphorylation level. A higher level of AS160 Thr^642^ phosphorylation at 3–4 h after exercise was also reported in some previous in vitro studies [23,24,25]. However, while previous studies with insulin treatment in vitro [23,24,25,41]), ex vivo [42], and hyperinsulinemic euglycemic clamp test in humans [43,44] at 3–4 h after exercise induced further AS160 Thr^642^ phosphorylation in skeletal muscle, we did not observe any additive effects of nutrient intake on AS160 Thr^642^ phosphorylation during the post-exercise phase in our experiments. In the present study, the plasma insulin concentration was increased at 15 min after nutrient treatment but returned to the pre-treatment level at 30 min. The changes in insulin level during the post-nutrient intake phase might have led to the different of AS160 phosphorylation results compared to the previous in vitro, ex vivo, and hyperinsulinemic clamp studies.

Akt is known as a central regulator of insulin-stimulated glucose uptake [45]. It is also known as the upstream kinase of AS160 [26]. The Thr^308^ residue of Akt is phosphorylated by 3-phosphoinositide-dependent protein kinase-1 (PDK1) and the Ser^473^ residue is phosphorylated by mechanistic target of rapamycin 2 (mTORC2) in insulin-dependent activation [46]. Both Thr^308^ and Ser^473^ phosphorylation are needed for the full activation of Akt [47]. We observed a positive main effect of the nutrient intake on Akt Thr^308^ phosphorylation was found in the plantaris muscle during the post-exercise phase. On the other hand, phosphorylation of Akt Ser^473^ was increased by the nutrient intake immediately after the exercise, but nutrient intake at 180 min after the exercise did not increase Akt Ser^473^ phosphorylation. Possibility exists that the absence of further AS160 phosphorylation after the nutrient intake at 180 min after the exercise was partly due to the impairment of Akt Ser^473^ phosphorylation. Moreover, a previous study using adipocytes suggested that there are Akt-dependent events other than AS160 regulation for GLUT4 trafficking to the plasma membrane [45,46,48], while whether such a pathway exists in skeletal muscle has not been fully clarified. Thus, the possibility exists that Akt activation might affect glucose uptake both AS160-dependent and independent manner. Although further studies should be needed to elucidate the significance of small changes in Akt phosphorylations, it can be thought the phosphorylated levels at the regulatory sites of Akt after the nutrient intake might influence on post-exercise skeletal muscle glycogen accumulation.

AMPK stimulates GLUT4 translocation to the plasma membrane with muscle contraction and then is considered a potential activator for glucose uptake during the post-exercise phase [13,49]. It is suggested that increased AS160 phosphorylation in response to insulin after exercise is facilitated by prior AMPK activation [50,51]. TBC1D1 also has the target site of AMPK (Ser^231^) and has been implicated to play a role in the GLUT4 translocation in response to muscle contraction [52,53,54]. In the present study, positive main effects of a 180 min fast after exercise were found in both phosphorylation levels of AMPK and ACC, the downstream protein substrate of AMPK. Consistent with the results of AMPK and ACC phosphorylations, there were significant positive main effects of time on AS160 Thr^642^ and TBC1D1 Ser^231^ phosphorylations. However, higher phosphorylation levels in AMPK signaling pathways did not result in higher glycogen repletion in the plantaris muscle. This result is consistent with our previous study showing that greater glycogen repletion induced by post-exercise ketone body administration was accompanied with lower phosphorylation levels in AMPK and ACC [31]. Other studies also reported that post-exercise enhancement of glucose transport with insulin treatment at 3 h after the exercise occurred without increased AMPK activation at the same time point [23,27]. Moreover, pharmacological activation of AMPK by 5-aminoimidazole-4-carboxamide ribonucleoside (AICAR) treatment did not result in a higher skeletal muscle glycogen level despite increased glucose uptake [55,56]. Collectively, these results suggest that persistent AMPK activation might not be necessary to enhance glycogen repletion during the post-exercise phase. Recent study using muscle-specific AMPK conditional knockout mice suggested that AMPK plays an important role on exercise-stimulated glycogen accumulation in skeletal muscle [43]. However, in that study, the human experiment showed that exercise induced various changes related to post-exercise glycogen accumulation other than AMPK activation; increased GLUT4 and hexokinase expressions, increased GS fractional activity, and greater phosphorylation (i.e., inactivation) of pyruvate dehydrogenase. Although AMPK activation might partly influence on post-exercise glycogen accumulation in skeletal muscle, post-exercise glycogen accumulation can be also affected by other factors.

Activation of GS is another important factor for the stimulation of glycogen repletion during the post-exercise phase. The GS affinity for G6P, the substrate for glycogen synthesis and the allosteric activator of GS, is increased by dephosphorylation of regulatory sites [57,58]. In particular, the phosphorylation level of GS Ser^641^ was shown to have a strong inverse correlation with GS fractional activity [58]. In the present study, GS Ser^641^ became significantly lower in response to nutrient intake immediately after the exercise. GS Ser^641^ phosphorylation in the LN and LC group was also lower than that in the EC group, but LN treatment did not induce further dephosphorylation of GS Ser^641^. Dephosphorylation of GS Ser^641^ would already have fully occurred during the 180 min fasting state after exercise.

We observed a significant positive effect of the 180 min fast after exercise for liver glycogen concentration. The increase in liver glycogen after 180 min from the end of exercise despite the no-food provision might have been due to the activation of gluconeogenesis. Meanwhile, we did not find a significant main effect of the nutrient intake. After exercise, exogenous glucose was preferentially utilized for skeletal muscle glycogen repletion rather than liver glycogen repletion [59,60]. A previous study showed in healthy rats that only 9% of infused glucose is used for hepatic glycogen synthesis after exhaustive exercise [61]. Therefore, it seems reasonable that post-exercise nutrient intake did not result in a significant increase in liver glycogen in the present study.

In a previous study, the plasma FFA concentration was elevated over the 2 h period of fasting following exercise [8]. Because an elevated blood FFA level has been reported to reduce glucose uptake and glycogen synthesis [33,34,35], we examined whether a 180 min fast after exercise affected the plasma FFA levels in our experiments. However, we found no main effect of the 180 min fast during the post-exercise phase. Therefore, in the present study, the plasma FFA level could not account for the impairment of glycogen accumulation by the 180 min fast after exercise.

## 5. Conclusions

Nutrient (carbohydrate and protein) intake immediately after an endurance exercise in the fed state significantly increased plantaris muscle glycogen concentration, while nutrient intake at 180 min after exercise appeared to have no effect on skeletal muscle glycogen accumulation in mice. While previous studies revealing a relationship between nutrient intake timing and post-exercise glycogen recovery after severe exercise depleting glycogen storage (e.g., exercise to exhaustion or with overnight fasting) [8,9,10], this is the first study demonstrating nutrient intake timing also affects skeletal muscle glycogen accumulation after exercise without severe glycogen depletion. Moreover, although some signaling proteins (AS160, AMPK, TBC1D1) related to exercise-dependent increase in glucose uptake were more phosphorylated after the 180 min period of fasting after exercise, these results were not associated with glycogen accumulation. Meanwhile, as significant increase in Akt Ser^473^ phosphorylation was induced only by the nutrient intake immediately after exercise, the possibility exists that Akt activation might have a role on post-exercise glycogen accumulation in skeletal muscle.
